# Food craving: new contributions on its assessment, moderators, and consequences

**DOI:** 10.3389/fpsyg.2015.00021

**Published:** 2015-01-22

**Authors:** Boris C. Rodríguez-Martín, Adrian Meule

**Affiliations:** ^1^Department of Psychology, Faculty of Psychology, Central University “Marta Abreu” of Las VillasSanta Clara, Cuba; ^2^Institute of Psychology, University of WürzburgWürzburg, Germany; ^3^Hospital for Child and Adolescent Psychiatry, LWL University Hospital of the Ruhr University BochumHamm, Germany

**Keywords:** food, cravings, emotional eating, obesity, pregnancy, binge eating, food addiction

Food craving refers to an intense desire to consume a specific food. In Western societies, these foods usually have high palatability and are energy dense, that is, they have high sugar and/or fat content. Food craving is a multidimensional experience as it includes cognitive (e.g., thinking about food), emotional (e.g., desire to eat or changes in mood), behavioral (e.g., seeking and consuming food), and physiological (e.g., salivation) aspects (Nederkoorn et al., 2000; Cepeda-Benito et al., 2000a).

Subjective self-report appears to be the most viable method for the assessment of craving as other measurement modalities (e.g., peripheral autonomic responses) typically suffer from a lack of specificity (Shiffman, 2000). The most often used instruments are the *Food Cravings Questionnaires* (FCQs; Cepeda-Benito et al., 2000a,b). Momentary food craving can be measured with a 15-item state version (FCQ-S) while the frequency of food craving experiences can be measured with a 39-item trait version (FCQ-T), which contains nine subscales. However, factor structure could not be replicated in some studies and as the FCQ-T usually has very high internal consistency, researchers often report its total score only. Consequently, such a long measure may not be necessary in order to assess a general index of trait food craving. In the current research topic, Meule et al. (2014) present a reduced version of the German FCQ-T (FCQ-T-r), which consists of 15 items only. Results showed that correlates of this short version were similar to those that have been found for the long version, for example, that FCQ-T scores are able to predict food-cue induced craving (Meule et al., 2012a,b). Following up on this, Rodríguez-Martín and Molerio-Pérez (2014) also could not replicate the nine-factorial structure of the Spanish FCQ-T (Cepeda-Benito et al., 2000b). They could demonstrate, however, that scores of the Spanish FCQ-T-r were highly correlated with scores of the long version as well as with the 24 excluded items. Moreover, it was shown that validity indices were similar for both versions, providing further support that the FCQ-T-r represents an adequate alternative for the assessment of trait food craving as measured by the FCQ-T.

Animal models are an important part of research on eating behavior. While people can be asked if they experience a desire to eat a food, measuring food craving in animals is not straightforward. Following abstinence from sugar, rats will exhibit a larger binge than ever before, which may be interpreted as an experience of craving (Avena et al., 2005). A rather indirect measure of food craving may be sensory-specific satiety (SST; specifically, the lack thereof). It refers to a temporary decline in food liking and food wanting derived from consuming a certain food in comparison to other unconsumed foods (Havermans et al., 2009). Reichelt et al. (2014) present a study in which rats consumed a so-called cafeteria diet of palatable, high-calorie foods for 2 weeks. They found that these rats showed impaired SST following consumption of a high-calorie solution, which may suggest that exposure to obesogenic diets impacts upon neurocircuitry involved in motivated control of eating behavior.

The ingestion of food is associated with a rewarding consequence and, thus, the incentive value of that particular food increases and its sensory attributes become signals for satisfaction (Havermans, 2013). Therefore, through Pavlovian conditioning, exposure to food-cues can likely trigger food craving. In experimental research, this can easily be examined by presenting pictorial food stimuli on a computer screen while psychophysiological data or subjective ratings are recorded. However, food image sets vary considerably across laboratories and image characteristics and food composition are often unspecified. Moreover, study results may be adversely affected by confounding variables such as perceptual characteristics of the stimuli. To remedy this, Blechert et al. (2014) developed a comprehensive database of food and non-food images along with detailed meta-data. This database will facilitate standardization and comparability across studies and will advance experimental research on food craving and eating behavior as it enables to match and control stimulus sets on a range of important variables.

Exposure to tempting food-cues, however, does not trigger food craving or lead to food consumption in each and every situation. Grubliauskiene and Dewitte (2014), for example, show that boys actually ate fewer candies following an unobtrusive pre-exposure to candies as compared to when there was no pre-exposure. Unexpectedly, however, this effect could not be shown for girls. Moreover, as was firstly demonstrated by Morewedge et al. (2010), instead of inducing food craving (i.e., having a sensitizing effect), repeatedly imagining the consumption of a food leads people to habituate to it and consequently reduces consumption of that food. Missbach et al. (2014) were able to replicate this finding with different food items than were used in the original studies. Importantly, they found that this habituation effect was neutralized by self-regulatory depletion. That is, repeated imagination of food consumption only reduces subsequent food intake when self-regulatory resources are available.

Research consistently demonstrates gender differences in food craving: women are more likely to experience food cravings than men (Weingarten and Elston, 1991). It is tempting to assume that these differences are related to hormonal differences between women and men, particularly as many women experience increases in food cravings perimenstrually and prenatally (Hormes, 2014). However, research on these experiences is scarce. Orloff and Hormes (2014) review the available literature on food cravings during pregnancy. They challenge the notion that perimenstrual or prenatal food craving is associated with hormonal changes, but suggest that cultural and psychosocial factors are more important determinants of food craving experiences during pregnancy and of excess gestational weight gain.

Food craving can occur as a result of specific mood states (often negative mood) and is marked by anticipation of mood enhancing effects of food intake (Cepeda-Benito et al., 2000a). Food craving is also associated with external eating, that is, it is often triggered by cues in the environment. Pfattheicher and Sassenrath (2014) report that emotional eating is positively related to individual differences in prevention focus while external eating is positively related to individual differences in promotion focus. Hence, this study showed that trait-like self-regulatory orientations are differentially related to specific eating styles, which may inspire intervention approaches for the reduction of food craving.

Potenza and Grilo (2014) briefly summarize contemporary research on food craving such as its neuronal underpinnings. They also highlight its relevance in obesity and binge eating disorder and suggest that research on and therapy of these disorders may benefit from providing an addiction framework. For instance, some approaches that effectively target drug cravings have also been shown to reduce food cravings. In a similar vein, Davis et al. (2014) investigated the effects of a methylphenidate challenge in individuals exhibiting addiction-like eating behavior. Individuals with “food addiction” reported more intense food craving than controls and were resistant to the food intake suppression that is typically induced by dopamine agonists. This supports that compulsive overeating is related to increased food craving and dopamine signaling-strength differences.

## Conflict of interest statement

The authors declare that the research was conducted in the absence of any commercial or financial relationships that could be construed as a potential conflict of interest.
